# Correlating natural language processing and automated speech analysis with clinician assessment to quantify speech-language changes in mild cognitive impairment and Alzheimer’s dementia

**DOI:** 10.1186/s13195-021-00848-x

**Published:** 2021-06-04

**Authors:** Anthony Yeung, Andrea Iaboni, Elizabeth Rochon, Monica Lavoie, Calvin Santiago, Maria Yancheva, Jekaterina Novikova, Mengdan Xu, Jessica Robin, Liam D. Kaufman, Fariya Mostafa

**Affiliations:** 1 Department of Psychiatry, University of Toronto, 250 College Street, Toronto, ON M5T 1R8 Canada; 2 KITE Research Institute, Toronto Rehab, University Health Network, Toronto, Canada; 3 Department of Speech-Language Pathology and Rehabilitation Sciences Institute, University of Toronto, Toronto, Canada; 4 Division of Neurology, Department of Medicine, University of Toronto, Toronto, Canada; 5 Winterlight Labs, Toronto, Canada

**Keywords:** Natural language processing, Automated speech analysis, Markers, Machine learning, Alzheimer’s, Dementia, Mild cognitive impairment

## Abstract

**Background:**

Language impairment is an important marker of neurodegenerative disorders. Despite this, there is no universal system of terminology used to describe these impairments and large inter-rater variability can exist between clinicians assessing language. The use of natural language processing (NLP) and automated speech analysis (ASA) is emerging as a novel and potentially more objective method to assess language in individuals with mild cognitive impairment (MCI) and Alzheimer’s dementia (AD). No studies have analyzed how variables extracted through NLP and ASA might also be correlated to language impairments identified by a clinician.

**Methods:**

Audio recordings (n=30) from participants with AD, MCI, and controls were rated by clinicians for word-finding difficulty, incoherence, perseveration, and errors in speech. Speech recordings were also transcribed, and linguistic and acoustic variables were extracted through NLP and ASA. Correlations between clinician-rated speech characteristics and the variables were compared using Spearman’s correlation. Exploratory factor analysis was applied to find common factors between variables for each speech characteristic.

**Results:**

Clinician agreement was high in three of the four speech characteristics: word-finding difficulty (ICC = 0.92, p<0.001), incoherence (ICC = 0.91, p<0.001), and perseveration (ICC = 0.88, p<0.001). Word-finding difficulty and incoherence were useful constructs at distinguishing MCI and AD from controls, while perseveration and speech errors were less relevant. Word-finding difficulty as a construct was explained by three factors, including number and duration of pauses, word duration, and syntactic complexity. Incoherence was explained by two factors, including increased average word duration, use of past tense, and changes in age of acquisition, and more negative valence.

**Conclusions:**

Variables extracted through automated acoustic and linguistic analysis of MCI and AD speech were significantly correlated with clinician ratings of speech and language characteristics. Our results suggest that correlating NLP and ASA with clinician observations is an objective and novel approach to measuring speech and language changes in neurodegenerative disorders.

**Supplementary Information:**

The online version contains supplementary material available at 10.1186/s13195-021-00848-x.

## Background

Language impairment is a core feature of Alzheimer’s disease (AD) and other neurodegenerative disorders [1]. Prior studies have shown a link between AD symptom severity and declining speech and language capability in picture description tasks [2–4]. Speech and language changes include alterations in speech rate, utterances, frequency of words, word-finding difficulties, and repetitions [5]. Despite these pervasive language changes, there is no universally accepted system of terminology used to describe language impairment, and large inter-rater variability can also exist between clinicians [6, 7].

Historically, rating scales have been developed to try to provide more objectivity [8]. For example, batteries include the Amsterdam-Nijmegen Everyday Language Test (ANELT), which assesses communicative abilities in patients with aphasia [9], the Boston Diagnostic Aphasia Examination (BDAE), and the Western Aphasia Battery (WAB), of which the last two assess for language and communication in stroke and AD patients [10, 11]. Although these can aid in standardizing clinician assessments, they are rarely utilized in daily clinical practice. One reason is that despite attempts at standardization, there remains inherent subjectivity with any human-based rating. For example, the commonly used clinical term “word-finding difficulty” is a non-specific clinical descriptor that spans across different diagnoses with different pathophysiological etiologies [12]. It is also variably defined between different health professions (e.g., family physicians, psychiatrists, neurologists, and speech language pathologists). Additionally, these batteries can be time-consuming and require training prior to administration.

In view of current limitations, technologies such as natural language processing (NLP) and automated speech analysis (ASA) are emerging as a novel, and potentially more objective method of assessing speech and language in individuals with neurologic and psychiatric disorders. In schizophrenia, use of NLP techniques such as latent semantic analysis can identify features such as incoherence [13]. In neurodegenerative disorders, ASA and NLP of vocal tasks have been demonstrated to be reliable markers for mild cognitive impairment (MCI) and AD [14, 15]. In patients with primary progressive aphasia (PPA), similar methods have enabled better identification of PPA variants, and the specific speech characteristics of each variant [16].

Despite these advances, no studies have investigated whether these extracted and analyzed variables have any relationship with clinician-rated characteristics. The ability to correlate NLP and ASA-extracted variables to clinician observations could be an important advancement in speech assessment and the diagnosis of neurodegenerative disorders. This has the potential to be a significant improvement over current methods by reducing assessment time, improving the reliability of impairment findings, and reducing clinician subjectivity.

Therefore, we sought to (1) define a set of speech and language characteristics that can be used by multidisciplinary clinicians; (2) determine if these characteristics are applied and rated consistently by clinicians in a sample of healthy controls (HC), MCI, and AD participants; (3) identify whether there are distinct differences in speech and language characteristics between these clinical groups; and (4) determine if linguistic and acoustic variables extracted through ASA and NLP have shared factors and can be correlated with clinician ratings.

## Methods

### Dataset

Speech recordings were obtained via the DementiaBank dataset through the TalkBank Project. The data were recorded as part of the Alzheimer’s Research Program at the University of Pittsburgh [17]. The dataset also contains demographics, diagnosis, and Mini-Mental Status Exam (MMSE) test scores from HC, MCI, and possible or probable AD participants [17]. At each annual visit, participants provided a speech recording which consists of a verbal description of the “Cookie Theft” picture from the BDAE. All participants also completed the MMSE on assessment. Data collection was approved by local institutional review boards, and all participants provided informed consent.

Clinical diagnoses were based on diagnoses assigned in DementiaBank, which were established after a comprehensive assessment including neuropsychological, medical, neurological, and psychiatric examination. In the original data set, the accuracy of the baseline clinical diagnosis relative to neuropathology was 86%, and when follow-up clinical data were considered, it reached 91.4% [17].

We applied an inclusion criterion of a minimum education level of 12 years or more, then randomly selected an equal number of speech samples from HC, MCI, and AD participants, for a total of 30 speech samples for the study. Clinical Dementia Rating (CDR) scores were obtained for each participant. Each speech sample was selected from a unique participant, except for one case in the MCI group in which two samples were chosen from the same participant since there were not enough unique female participants with MCI in the dataset. All healthy control participants had an MMSE score of 27 or higher, MCI participants had an MMSE score between 23 and 26, and AD participants had an MMSE score between 15 and 20 [17].

### Defining speech and language characteristics

The speech recordings were rated by 5 clinicians (1 geriatric psychiatrist, 1 psychiatry resident, 1 neurology resident, and 2 speech language pathologists) with prior clinical experience in speech and language assessment of patients with MCI and AD. Clinician selection was done through internal recruiting of clinicians previously affiliated with speech research at our institution. Prior to assessing the speech recordings, a group consensus approach and literature review was used to select four clinically-relevant speech and language characteristics to rate (Table 1). The four characteristics chosen were (1) word-finding difficulty, (2) incoherence, (3) perseveration, and (4) errors in speech. These characteristics were chosen because of their relevance in MCI and AD and relevance to clinical descriptors in the mental status examination [18]. A consensus rubric was created for clinicians to rate each characteristic on a Likert scale (range 0–3) as being not present or normal finding (0), mild (1), moderate (2), or severe (3).

**Table 1:** Clinician consensus table of speech and language characteristics

Characteristic	Clinical features
**Word-finding difficulty**	● Reduction in content words, circumlocution, and false starts [19] ● Pauses while searching for words [12] ● Fluency (rate, phrase length, amount of hesitation) ● Revisions (repetitions of complete words or phrases/elaborations), and indefinite terms (fillers)
**Incoherence**	● Coherence is the orderly flow of information within discourse (graph features), and a marker of how well discourse is connected within words, sentences, and overall speech (local and global coherence) [20–23] ● Incoherence is characterized by disorganized speech, derailment or sudden topic shifts, tangentiality, flight of ideas, or word salad [13, 18, 24, 25].
**Perseveration**	● Repetition of word or phrase even after the stimulus for the behavior (word or phrase) has been taken away [18, 26] ● Persistence of behavior (word or phrase) despite repeated failure ● Intrusion: inappropriate repetition of prior responses after intervening stimuli [27]
**Errors in Speech**	● Phonetic errors (omissions, additions, substitutions, distortions) [28] ● Stuttering [18] ● Sequences of phonemic approximation

Clinicians then independently rated each speech recording and were blind to the diagnostic labels. For the majority of ratings (142 out of 150), rating discrepancies between clinicians were within ±1, and the modal value was established as the group consensus rating. There were 8 items, from 4 recordings, where the rating discrepancy was ±2. These samples were much shorter in length or had poorer audio quality. In these cases, clinicians were asked to re-rate the items. After re-rating, the rating discrepancies were within ±1 and the consensus rating was established using the modal value.

### Lexical, semantic, syntactic, and acoustic variable extraction

The speech recordings were transcribed, and annotations such as speaker segmentation and utterance segmentation were generated by trained raters using customized transcription software. NLP-extracted variables included lexical (e.g., rates and types of words used, and their characteristics such as frequency or age of acquisition), semantic (e.g., semantic relatedness of subsequent utterances, semantic relatedness of utterances to the items in the picture), and syntactic (e.g., syntactic complexity, use of different syntactic constructions) aspects of the recording. Acoustic variables (e.g., properties of the sound wave, speech rate, number of pauses) were extracted using ASA. Data processing and feature extraction were performed automatically using a combination of Python-based standard acoustic and language processing libraries (e.g., spaCy), and customized code. For each speech recording, a total of 540 variables were computed based on the sound file and accompanying transcript.

### Inter-rater reliability of clinician ratings

To determine the consistency of the clinicians’ ratings of speech and language characteristics, intra-class correlations (ICC) were calculated [29]. Interpretations of ICC results were based on previously published guidelines (0.50 for poor, 0.5–0.75 for moderate, 0.75–0.90 for good, and >0.90 for excellent agreement) [30].

### Exploratory factor analysis of speech characteristics

Odds ratios (OR) were calculated for each characteristic and compared between the clinical groups. Spearman correlation tests were conducted between variables extracted and the consensus clinician ratings. For each of the four characteristics, variables with significant correlations (p<0.05) underwent exploratory factor analysis (EFA). EFA was performed following guidelines by Fabrigar et al. on factor extraction procedure, factor number, and rotation of factors [31]. Principal factor extraction was conducted and parallel analysis (PA) was applied to determine the number of factors for each characteristic. Factors with eigenvalues greater than 95th percentile of PA eigenvalues from 100 iterations are suggested for retention. Oblique rotation was adopted as the factor rotation method to allow for correlation among latent factors, following the methods used in Fraser et al. [31, 32]. In addition, due to our small sample size, we chose to set a conservative factor loading cutoff of 0.6 [33]. Statistical analyses were conducted using R 3.6.3 and Python 3.6 [34].

## Results

Participant demographics are described in Table 2. The average age of the participants was 65.0 years, with equal numbers of samples from males and females.

**Table 2:** Sample demographics by diagnostic group

	HC (n=10)	MCI (n=10)	AD (n=10)
**Age at visit, mean (SD), years**	61.2 (9.67)	69.9 (5.85)	64.0 (10.99)
**Female (%)**	50	50	50
**MMSE, mean (SD)**	29 (0.89)	24 (1.95)	18 (1.60)
**CDR, mean (SD)**	0.05 (0.16)	0.75 (0.26)	1.1 (0.32)
**Education, mean (SD), years**	14.2 (2.29)	14.0 (1.86)	13.8 (2.17)

### Clinician agreement of speech and language characteristics

Clinician agreement was high for word-finding difficulty (ICC=0.92, p<0.001), incoherence (ICC=0.91, p<0.001), and perseveration (ICC=0.88, p<0.001). Errors in speech had moderate agreement (ICC=0.67, p<0.001). Since there was consistent agreement between clinician raters, the overall clinician consensus rating was used in subsequent analyses.

**Fig. 1:**
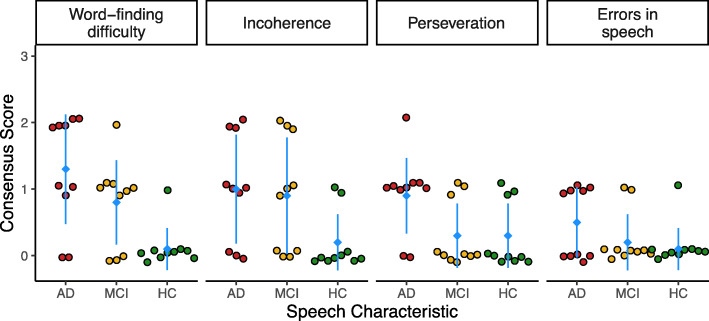
Distribution of the consensus clinician ratings for each speech and language characteristic, by diagnosis group. The mean consensus rating for each group is indicated with a blue diamond and whiskers indicate the standard deviation. For all ratings, a rating of 3 = severe, 2 = moderate, 1 = mild, and 0 = no presence or a normal finding of that characteristic. AD Alzheimer’s dementia, MCI mild cognitive impairment, HC healthy control

Clinician ratings differed between the AD, MCI, and control participants (Fig. 1). Ratings were generally the highest (greatest impairment) in the AD group, followed by MCI and controls. In particular, the odds of impairment in word-finding were higher in AD (OR 68.0, 95%, 6.9–1741.2) and MCI (OR 16.8, 95%, 2.1–368.7) compared to controls, with no difference found between AD and MCI (OR 4.1, 95%, 0.7–27.3). The odds of incoherence were also higher in AD (OR 9.5, 95%, 1.5–86.0) and MCI (OR 7.4, 95%, 1.2–67.2) compared to controls, with no difference between AD and MCI (OR 1.3, 95%, 0.3–6.8). The odds of impairment in perseveration were higher in AD compared to both MCI (OR 10.4, 95%, 1.5–104.4) and controls (OR 10.4, 95%, 1.5–104.4), with no difference between MCI and controls (OR 1.0, 95%, 0.1–7.1). Finally, the odds of increased errors in speech were higher for AD compared to controls (OR 9.0, 95%, 1.1–200.4), with no difference between AD and MCI (OR 4.0, 95%, 0.6–36.5) or between MCI and controls (OR 2.3, 95%, 0.2–54.0).

### Word-finding difficulty

A total of 31 variables were correlated with ratings of word-finding difficulty (p<0.05; Table 3). The variables with the highest correlations to the clinician ratings were related to rate of speech, word duration, and length and the number of unfilled (silent) pauses. Greater severity of word-finding difficulty was associated with slower speech, shorter words, and increased pauses. Four factors were identified in the EFA (Table 3). The first factor (15.3% of the total variance) included variables relating to the number and duration of pauses. The second factor (12.9% variance) included word duration, acoustic variables including characteristics of Mel-frequency cepstral coefficients (MFCCs), and the use of different types of noun and verb phrases. The third factor (12.9% variance) included variables relating to the zero-crossing rate of speech (ZCR). MFCCs and ZCR represent mathematical (spectral) properties of a sound wave and acoustic features of speech. MFCCs are coefficients that make up the Mel-frequency cepstrum, the short-term power spectrum of a sound [35]. ZCR indicates the rate of sign changes of a signal and can be used to measure frequency. The fourth factor (11.5% variance) included measures of word length, word frequency, total number of words produced, and syntactic complexity, as measured by the number of coordinate phrases per clause.

**Table 3:** Word-finding difficulty (clinician-rated) and correlations (p<0.05) with speech variables, ranked by correlation coefficient (Spearman’s |ρ|)

Variable type	Variable description	ρ	F1	F2	F3	F4
Lexical	Average word length	− 0.74				0.78
Acoustic	Speech rate	− 0.68				
Acoustic	Average word duration	0.68		0.62		
Acoustic	Number of unfilled pauses	0.63	− 0.68			
Acoustic	Number of long pauses	0.48	− 0.87			
Lexical	Use of past tense verbs	0.47				
Acoustic	Variance of the number of zero crossings	− 0.46			− 0.81	
Acoustic	Skewness of the number of zero crossings	0.46			0.91	
Acoustic	Mean number of zero crossings	− 0.46			− 0.79	
Acoustic	Kurtosis of the number of zero crossings	0.46			0.91	
Acoustic	Total duration of short pauses	− 0.46	0.63			
Lexical	Ratio of subordinate to coordinate words	0.46				
Lexical	Average word frequency	0.43				− 0.79
Acoustic	Total duration of long pauses	0.43	− 0.87			
Syntactic	Use of noun phrases with determiners and nouns	0.42		0.67		
Acoustic	Number of short pauses	− 0.42	0.72			
Syntactic	Use of conjunctive verb phrases	− 0.41				
Acoustic	Mean pause duration	0.39	− 0.66			
Lexical	Use of demonstrative words	0.39				
Syntactic	Use of singular present verb phrases with prepositional phrases	− 0.39				
Syntactic	Number of coordinate phrases per clause	− 0.39				0.72
Lexical	Number of words	0.39				− 0.64
Syntactic	Use of coordinate phrases	− 0.38				
Acoustic	Skewness of the second derivative of the 4th MFCC	− 0.38		− 0.85		
Lexical	Average dominance score of all words	− 0.37				
Acoustic	Kurtosis of the second derivative of the 4th MFCC	0.37		0.94		
Lexical	Use of comparative adjectives	0.37				
Syntactic	Use of past tense verb phrases with noun phrases	0.37		0.73		
Acoustic	Mean of the first derivative of the 10th MFCC	− 0.37				
Lexical	Average dominance scores of nouns	− 0.36				
Syntactic	Use of adjective phrases	− 0.36				

Columns F1 to F4 indicate which variables were assigned to each factor and the factor loading scores. Variables that do not have values in any of the columns correlated with word-finding difficulty but were not included in a factor based on the EFA

### Incoherence

For incoherence, a total of 20 variables were correlated (p<0.05; Table 4). Variables with the highest correlations included a mix of syntactic, acoustic and lexical variables, reflecting the use of past tense verb phrases, slower speech rate, and usage of words with higher estimated age of acquisition and more negative valence in the content of speech. Two factors were identified in the EFA (Table 4). The first factor (26.3% variance) included acoustic variables reflecting properties of the sound wave, word duration, and use of past tense verb phrases. The second factor (17.1% variance) included variables relating to the age of acquisition and valence of the words used.

**Table 4:** Incoherence (clinician-rated) and correlations (p<0.05) with speech variables, ranked by correlation coefficient (Spearman’s |ρ|)

Variable type	Variable description	ρ	F1	F2
Syntactic	Use of past tense verb phrases with noun phrases	0.51	0.69	
Lexical	Average age of acquisition score of all words	0.47		− 0.64
Acoustic	Speech rate	− 0.47		
Acoustic	Average word duration	0.47	0.64	
Lexical	Average age of acquisition score of nouns	0.46		− 0.65
Lexical	Average valence score of nouns	− 0.43		0.79
Acoustic	Skewness of the second derivative of the 4th MFCC	− 0.42	− 0.87	
Semantic	Proportion of subject words used	− 0.42		
Syntactic	Use of 3rd person singular present verb phrases with prepositional phrases	− 0.41		
Acoustic	Kurtosis of the second derivative of the 4th MFCC	0.41	0.98	
Syntactic	Use of prepositional phrases with noun phrases	− 0.40		
Acoustic	Skewness of the second derivative of the log energy	− 0.39	− 0.96	
Lexical	Use of comparative adjectives	0.38		
Acoustic	Kurtosis of the second derivative of the log energy	0.38	0.95	
Semantic	Semantic similarity of description to picture content (max cosine distance, 300-dim word vectors)	0.38		
Semantic	Average valence score of all words	− 0.37		0.82
Syntactic	Use of singular present verb phrases with prepositional phrases	− 0.37		
Syntactic	Use of verb phrases with noun phrases	0.37		
Semantic	Proportion of subjects in picture described	− 0.37		
Lexical	Average arousal score of nouns	− 0.36		

Columns F1 and F2 indicate which variables were assigned to each factor, and the factor loading scores. Variables that do not have values in columns F1 and F2 are correlated with incoherence but were not included in a factor based on the exploratory factor analysis

### Perseveration

For perseveration, a total of 93 variables were correlated (see Supplementary Table S1). The variables with the highest correlations included measures relating to the repetitiveness of utterances (measured using cosine distance between utterances), the semantic similarity of utterances to the items in the picture (measured with word embedding models of semantic similarity), and vocabulary richness (measured by the diversity of words used). We measured the repetitiveness of utterances by representing the words used as vectors and calculating the cosine distance between pairs of utterances based on the overlap in word usage. Speech with a low average cosine distance value represents increased repetition of the same words across different utterances. Utterances that use different words, even if remaining on a similar topic, would have a high cosine distance. In addition, we used word embedding models to assess the semantic similarity of the utterances to key words in the picture. In this case, the maximum cosine distance was positively associated with perseveration scores, indicating that those with higher perseveration ratings had utterances that were more dissimilar to the picture key words than those with lower perseveration ratings. Thus, the greater severity of perseveration was associated with increased repetitiveness of speech, decreased vocabulary richness, and decreased semantic similarity of a participant’s speech to the items in the picture described. A large number of acoustic variables (MFCC variables) also correlated with perseveration. A single factor was identified in the EFA (see Supplementary Table S1), explaining 72.9% of the variance. This factor was primarily made up of acoustic variables, measures of audio duration, and vocabulary richness.

### Errors in speech

For errors in speech, a total of 49 variables were correlated (see Supplementary Table S2). Variables with the highest correlations included measures relating to the complexity of speech and vocabulary, use of subordinate clauses, and word length. Greater severity of errors in speech was associated with decreased complexity of speech as measured by graph metrics (the organization of speech using graph network analysis), decreased vocabulary richness (measured by the diversity of words used), use of shorter words, and increased use of verb phrases with subordinate clauses. The use of verb phrases with subordinate clauses was found to frequently occur for utterances that contained sentence fragments or ungrammatical constructions (e.g., “It’s -- she doesn’t seem to think it’s even know what's going on”). A total of two factors were identified in the EFA (see Supplementary Table S2). The first factor (35.1% of the variance) included solely acoustic variables (i.e., MFCC variables), and the second factor (18.8% of the variance) included variables relating to vocabulary richness and complexity of speech.

## Discussion

In this exploratory study, we first examined whether multidisciplinary clinicians could rate a set of predefined speech and language characteristics consistently in a sample of controls, MCI, and AD participants. We also examined whether linguistic and acoustic variables extracted through NLP and ASA correlated with these clinician ratings.

First, the ICC ratings demonstrated good agreement and consistency between clinicians for the characteristics of word-finding difficulty, incoherence, and perseveration. This demonstrates that despite inherent subjectivity in assessing speech, consensus can be reached across multidisciplinary clinicians. Our results demonstrated greater severity of word-finding difficulty and incoherence in both MCI and AD compared to controls. This finding is consistent with the clinical speech changes seen in MCI and AD, which include impairments in fluency, confrontational naming, and increased repetition of words [36, 37]. Thus, word-finding difficulty and incoherence may be particularly useful constructs to include when developing automated speech tools for MCI and AD.

Our correlation analysis between variables extracted from NLP and ASA with clinician ratings had several notable findings. Word-finding difficulty was explained by four clinically relevant factors: (1) variables relating to the number and duration of pauses; (2) word duration, MFCCs, and rate of noun and verb phrases; (3) ZCR variables; and (4) word length, word frequency, total words, and syntactic complexity. Our findings are in line with previous NLP studies using the DementiaBank dataset, which have also found greater pauses and syntactic errors in AD participants [38]. Pauses have been hypothesized to be a compensatory mechanism in the earlier stages of AD [39], and our findings demonstrate that pauses are also a core feature of how clinicians defined word-finding difficulty. This finding has been replicated in other datasets in the MCI population as well [40]. The use of shorter words and more frequent words is well-characterized during picture description tasks in AD [5, 41], and increasing word frequency is also correlated to AD severity [2]. As noted previously, word-finding difficulty is variably defined between different health professions in the clinical setting [12]. However, our study found high consensus between clinicians, in addition to correlation with the extracted speech variables, which suggests that word-finding difficulty can be measured objectively and reliably.

Incoherence was explained by two factors: (1) increased average word duration, increased use of past tense verb phrases, and acoustic changes, and (2) words with higher estimated ages of acquisition and more negative valence in the content of speech. The finding of increased average word duration is consistent with previous speech studies where both AD and MCI were found to have increased average word and syllable duration [40, 42]. Clinically, this can present as either a slower speaking rate [43] or as hesitations in speech, both of which are well-defined characteristics in MCI and AD [44]. Our finding of the usage of past tense verbs, words with higher age of acquisition, and more negative valence may all represent deviations from describing the “Cookie Theft” picture. The picture description task contains objects with names that have low ages of acquisition (e.g., “boy,” “girl,” “water,” “plate”) and tends to be described in the present tense. Atypical and off-topic utterances would therefore lead to deviations in the types of words and sentences used, which would explain the relationship of words with higher age of acquisition, and more negative valence with higher incoherence ratings. We also note that the evidence remains mixed as to whether AD patients are more impaired than controls in past tense verb morphology in general [45].

In the remaining two characteristics, perseveration was explained by one factor (acoustic variables, measures of audio duration, and vocabulary richness), with the highest correlations relating to cosine distance of utterances, reflecting repetitive speech. Errors in speech were explained by two factors (vocabulary richness and complexity of speech, and another to acoustic changes), with the highest correlation with variables reflecting use of verb phrases with subordinate clauses, which reflect utterances with grammatical errors or incomplete utterances. We interpret the findings in these two characteristics with more caution, since both perseveration and errors in speech had the lowest ICC between clinicians. Additionally, they were mainly rated as being not present or mild. We hypothesize the length of recordings (typically around 1 min) may be too short for clinicians to adequately assess for the presence of these characteristics. Alternatively, AD and MCI may be less likely to produce speech errors, or these characteristics may only be evident in severe AD, which was not captured in this sample [46, 47]. When perseveration and speech errors were noted by clinicians, they tended to be in AD participants, and not MCI. Thus, the use of larger samples with broader ranges of impairment, and longer samples of speech, may be better able to shed light on the clinical utility of these two specific characteristics.

Currently, manual analysis of speech and language is affected by rater bias and differences in observational techniques [3, 37, 48]. Despite the “Cookie Theft” task being one of the most common research and clinical tools, a recent systematic review found several limitations in its current implementation and use [37]. One key limitation is the lack of cohesiveness in language impairment terminology between studies that analyze speech and language in this task. This limits the ability to aggregate results across studies and to objectively track pathologic changes over time. Another current limitation is the finite number of skilled and experienced clinicians who can complete these assessments reliably. Thus, an automated approach to assessing speech could serve as a highly scalable approach, compared to the time that would be required to train a clinician. To our knowledge, our study is the first to provide a proof-of-concept solution to these limitations by integrating clinician consensus with objective acoustic, lexical, semantic, and syntactic variables extracted through NLP and ASA. Our results show this approach provides a rational, objective, and clinically correlated way to characterize speech and language impairments in MCI and AD.

## Limitations

Limitations of our study include a small sample size of participants and rating clinicians, which limits generalizability of our findings. Accordingly, the estimated ORs for language impairments by clinical groups had large confidence intervals. Follow-up work with larger datasets will better quantify the odds of speech and language changes according to clinical status. As we had a small number of clinicians rating the speech samples, we cannot rule out the possibility of systematic biases in rating speech deficits, and our study is not powered to detect these differences.

In addition, since we only included English-speaking participants, it is unknown if the results are applicable across different languages. Although the DementiaBank corpus reported high accuracy (86%) between baseline clinical diagnosis relative to final neuropathologic diagnosis, the diagnosis of MCI represents a clinically heterogeneous population that includes non-Alzheimer's type pathology [17]. Thus, the MCI sample may not be clinically representative for individuals with the diagnosis outside of this study dataset.

One limitation of using the Cookie Theft picture description task is that some of the significant findings identified in this study may be only characteristic for the task itself. For example, higher usage of past tense verbs may indicate a deviation from the task, since pictures are usually described in present tense. Thus, future studies comparing standardized speech tasks versus spontaneous, conversational speech may help determine which language patterns are specific to the Cookie Theft task, and which are general changes that occur in all forms of speech.

One final limitation relates to the large set of extracted variables through NLP and ASA, which means that spurious associations cannot be ruled out. However, this has been mitigated by considering the clinical manifestations of MCI and AD and by referencing our positive findings to existing literature and previous analyses using the DementiaBank dataset [38].

## Conclusions

Currently, there remains an urgent need for markers of disease-specific language impairment in both prodromal and diagnosed Alzheimer’s disease [3]. Early identification of these markers could improve clinicians’ ability to distinguish AD from normal age-related changes. Our study provides evidence and validation that NLP and ASA can not only detect objective speech-language changes in MCI and AD, but that these changes can also be directly correlated to clinician assessment of speech. With further validation through larger datasets and a greater number of clinician raters, this approach may present as a novel method of clinical assessment and could also inform the development of digital speech and language markers as well. Other future areas of research include using larger datasets to develop standardized frameworks for natural language processing in neurodegenerative and psychiatric disorders. Our results serve as a proof-of-concept for using an automated, objective, and data-driven approach to define subjective clinical speech and language characteristics in neurodegenerative disorders.

## Supplementary Information

**Additional file 1:.** Supplemental Information. Supplemental Table S1 and Supplemental Table S2.

## Data Availability

The audio dataset analyzed from this study is available from the DementiaBank repository, https://dementia.talkbank.org/access [17]. The analysis dataset generated is available from the corresponding author on reasonable request.
