# Supplementary material for: Correlating natural language processing and automated speech analysis with clinician assessment to quantify speech-language changes in mild cognitive impairment and Alzheimer’s dementia
Source: Alzheimers Res Ther. 2021 Jun 4;13:109. doi: 10.1186/s13195-021-00848-x (PMC8178861; doi:10.1186/s13195-021-00848-x)
Supplement: Supplementary file 1 — Additional file 1:. Supplemental Information. Supplemental Table S1 and Supplemental Table S2. [file 13195_2021_848_MOESM1_ESM.docx]

##### **Supplemental Information**

##### **Supplemental Table S1.** Perseveration (clinician-rated) and correlations (p<0.05) with variables, ranked by correlation coefficient (Spearman’s |ρ|).

| **Variable type** | **Variable description** | **ρ** | **F1** |
| --- | --- | --- | --- |
| Semantic | Utterance repetition (pairs of utterances with cosine distance < 0.5) | 0.68 |  |
| Semantic | Utterance repetition (minimum cosine distance) | -0.55 |  |
| Lexical | Vocabulary richness (Honore’s statistics) | -0.55 |  |
| Semantic | Semantic similarity of description to picture content (max cosine distance, 300-dim word vectors) | 0.52 |  |
| Acoustic | Kurtosis of the second derivative of the 3rd MFCC | 0.50 | 0.89 |
| Syntactic | Syntactic complexity (average tree depth) | -0.50 |  |
| Acoustic | Skewness of the second derivative of the 8th MFCC | -0.50 | -1.00 |
| Lexical | Use of past tense verbs | 0.50 |  |
| Acoustic | Kurtosis of the first derivative of the 11th MFCC | 0.49 | 0.81 |
| Acoustic | Skewness of the second derivative of the 5th MFCC | -0.49 | -0.99 |
| Acoustic | Skewness of the first derivative of the 6th MFCC | 0.49 | 0.99 |
| Acoustic | Kurtosis of the second derivative of the 8th MFCC | 0.49 | 1.00 |
| Acoustic | Kurtosis of the second derivative of the 5th MFCC | 0.49 | 1.00 |
| Syntactic | Minimum length of utterance (MLU) | -0.49 |  |
| Acoustic | Kurtosis of the first derivative of the 6th MFCC | 0.49 | 0.99 |
| Acoustic | Total length of the audio sample in seconds | 0.48 | 0.99 |
| Acoustic | Skewness of the 9th MFCC | 0.48 | 0.99 |
| Acoustic | Skewness of the second derivative of the 6th MFCC | -0.48 | -0.99 |
| Acoustic | Skewness of the second derivative of the 2nd MFCC | 0.48 | 1.00 |
| Acoustic | Skewness of the 12th MFCC | 0.48 | 0.99 |
| Acoustic | Skewness of the 11th MFCC | 0.48 | 0.99 |
| Acoustic | Skewness of the 10th MFCC | 0.48 | 0.99 |
| Acoustic | Kurtosis of the 9th MFCC | 0.48 | 0.99 |
| Acoustic | Kurtosis of the 8th MFCC | 0.48 | 0.99 |
| Acoustic | Kurtosis of the second derivative of the 6th MFCC | 0.48 | 1.00 |
| Acoustic | Kurtosis of the second derivative of the 2nd MFCC | 0.48 | 1.00 |
| Acoustic | Kurtosis of the 12th MFCC | 0.48 | 0.99 |
| Acoustic | Kurtosis of the 11th MFCC | 0.48 | 0.99 |
| Acoustic | Kurtosis of the 10th MFCC | 0.48 | 0.99 |
| Acoustic | Skewness of the second derivative of the log energy | -0.48 | -0.98 |
| Semantic | Proportion of subject words used | -0.47 |  |
| Acoustic | Skewness of the 6th MFCC | 0.47 | 0.99 |
| Acoustic | Skewness of the 5th MFCC | 0.47 | 0.99 |
| Acoustic | Skewness of the 4th MFCC | 0.47 | 0.99 |
| Acoustic | Skewness of the second derivative of the 7th MFCC | -0.47 | -0.99 |
| Acoustic | Skewness of the 3rd MFCC | 0.47 | 0.99 |
| Acoustic | Skewness of the second derivative of the 1st MFCC | 0.47 | 0.99 |
| Acoustic | Skewness of the second derivative of the 0th MFCC | 0.47 | 0.99 |
| Acoustic | Skewness of the 2nd MFCC | 0.47 | 0.99 |
| Acoustic | Skewness of the first derivative of the 5th MFCC | 0.47 | 0.99 |
| Acoustic | Skewness of the first derivative of the 4th MFCC | 0.47 | 0.99 |
| Acoustic | Skewness of the first derivative of the 3rd MFCC | 0.47 | 0.99 |
| Acoustic | Skewness of the first derivative of the 2nd MFCC | 0.47 | 0.99 |
| Acoustic | Skewness of the first derivative of the 1st MFCC | 0.47 | 0.99 |
| Acoustic | Skewness of the first derivative of the 0th MFCC | 0.47 | 0.99 |
| Acoustic | Skewness of the 1st MFCC | 0.47 | 0.99 |
| Acoustic | Skewness of the 0th MFCC | 0.47 | 0.99 |
| Acoustic | Kurtosis of the 7th MFCC | 0.47 | 0.99 |
| Acoustic | Kurtosis of the 6th MFCC | 0.47 | 0.99 |
| Acoustic | Kurtosis of the 5th MFCC | 0.47 | 1.00 |
| Acoustic | Kurtosis of the second derivative of the log energy | 0.47 | 0.99 |
| Acoustic | Kurtosis of the 4th MFCC | 0.47 | 1.00 |
| Acoustic | Kurtosis of the second derivative of the 7th MFCC | 0.47 | 1.00 |
| Acoustic | Kurtosis of the 3rd MFCC | 0.47 | 1.00 |
| Acoustic | Kurtosis of the second derivative of the 1st MFCC | 0.47 | 1.00 |
| Acoustic | Kurtosis of the second derivative of the 0th MFCC | 0.47 | 0.99 |
| Acoustic | Kurtosis of the 2nd MFCC | 0.47 | 1.00 |
| Acoustic | Kurtosis of the first derivative of the 4th MFCC | 0.47 | 1.00 |
| Acoustic | Kurtosis of the first derivative of the 3rd MFCC | 0.47 | 1.00 |
| Acoustic | Kurtosis of the first derivative of the 2nd MFCC | 0.47 | 1.00 |
| Acoustic | Kurtosis of the first derivative of the 1st MFCC | 0.47 | 1.00 |
| Acoustic | Kurtosis of the first derivative of the 0th MFCC | 0.47 | 0.99 |
| Acoustic | Kurtosis of the 1st MFCC | 0.47 | 1.00 |
| Acoustic | Kurtosis of the 0th MFCC | 0.47 | 0.99 |
| Acoustic | Skewness of the 8th MFCC | 0.46 | 0.99 |
| Acoustic | Skewness of the first derivative of the log energy | -0.46 | -0.99 |
| Acoustic | Kurtosis of the first derivative of the 5th MFCC | 0.46 | 1.00 |
| Acoustic | Skewness of the 7th MFCC | 0.46 | 0.99 |
| Acoustic | Kurtosis of the first derivative of the log energy | 0.46 | 0.99 |
| Acoustic | Kurtosis of the second derivative of the 4th MFCC coefficient | 0.46 | 0.93 |
| Syntactic | Use of singular present verb phrases with declarative clauses | 0.44 |  |
| Lexical | Vocabulary richness (TTR, ratio of unique words to total number of words) | -0.43 | -0.68 |
| Acoustic | Skewness of the second derivative of the 3rd MFCC | 0.43 | 0.77 |
| Semantic | Complexity of speech (undirect parallel edges in graph) | 0.43 |  |
| Acoustic | Kurtosis of the first derivative of the 10th MFCC | 0.42 | 0.79 |
| Acoustic | Kurtosis of the first derivative of the 12th MFCC | 0.42 | 0.80 |
| Acoustic | Skewness of the second derivative of the 4th MFCC | -0.41 | -0.79 |
| Syntactic | Use of noun phrases with determiners and nouns | 0.41 |  |
| Acoustic | Skewness of the first derivative of the 11th MFCC | -0.41 | -0.73 |
| Lexical | Use of (existential) there | 0.41 |  |
| Syntactic | Use of subordinate clauses with wh-noun phrases and declarative clauses | -0.40 |  |
| Syntactic | Syntactic complexity (maximum tree depth) | -0.40 |  |
| Semantic | Complexity of speech (directed parallel edges in graph) | 0.40 |  |
| Lexical | Vocabulary richness (Brunet’s index) | 0.39 | 0.73 |
| Acoustic | Skewness of the first derivative of the 10th MFCC | -0.39 | -0.70 |
| Syntactic | Use of verb phrases with declarative clauses | 0.39 |  |
| Syntactic | Use of 3rd person singular present verb phrases with noun phrases | 0.39 |  |
| Acoustic | Kurtosis of the first derivative of the 9th MFCC | 0.38 | 0.78 |
| Semantic | Semantic similarity of successive utterances (max cosine distance, 200-dim word vectors) | 0.38 |  |
| Semantic | Semantic similarity of successive utterances (max cosine distance, 300-dim word vectors) | 0.38 |  |
| Syntactic | Use of verb phrases with subordinate clauses | 0.37 |  |
| Acoustic | Skewness of the second derivative of the 10th MFCC | -0.37 |  |
| Semantic | Semantic similarity of successive utterances (max cosine distance, 50-dim word vectors) | 0.36 |  |

*Column F1 indicates which variables were included in the factor and the factor loading scores. Variables that do not have values listed in column F1 correlated with perseveration but were not included in a factor based on the exploratory factor analysis.*

#####

##### **Supplemental Table S2.** Errors in speech (clinician-rated) and correlations (p < 0.05) with variables, ranked by correlation coefficient (Spearman’s |ρ|).

| **Variable type** | **Variable description** | **ρ** | **F1** | **F2** |
| --- | --- | --- | --- | --- |
| Syntactic | Use of 3rd person singular present verb phrases with subordinate clauses | 0.58 |  |  |
| Semantic | Complexity of speech (number of nodes in graph) | -0.51 |  | 0.75 |
| Semantic | Complexity of speech (number of nodes in the largest connected subgraph) | -0.51 |  | 0.75 |
| Lexical | Average word length | -0.50 |  |  |
| Acoustic | Variance of the log energy | 0.49 |  |  |
| Lexical | Vocabulary richness (TTR, ratio of unique words to total number of words, 50 word window) | -0.49 |  | 0.82 |
| Lexical | Ratio of subordinate to coordinate words | 0.48 |  |  |
| Lexical | Vocabulary richness (TTR, ratio of unique words to total number of words) | -0.46 |  | 0.74 |
| Semantic | Complexity of speech (length of longest shortest path between all node pairs) | -0.46 |  | 0.65 |
| Semantic | Complexity of speech (average length of the shortest path between pairs of nodes) | -0.46 |  | 0.63 |
| Semantic | Complexity of speech (number of nodes in the largest strongly connected subgraph) | -0.45 |  | 0.71 |
| Lexical | Vocabulary richness (TTR, ratio of unique words to total number of words, 40 word window) | -0.45 |  | 0.79 |
| Syntactic | Use of wh-adverb phrases (e.g., where, when, why) | 0.45 |  |  |
| Semantic | Semantic similarity of description to picture content (max cosine distance, 300-dim word vectors) | 0.44 |  |  |
| Syntactic | Use of noun phrases with cardinal numbers and nouns | 0.44 |  |  |
| Syntactic | Use of noun phrases with cardinal numbers | 0.44 |  |  |
| Acoustic | Mean of the 6th MFCC | -0.44 | 0.92 |  |
| Semantic | Proportion of location words used | -0.43 |  |  |
| Acoustic | Mean of the 11th MFCC | -0.43 | 0.92 |  |
| Syntactic | Use of declarative clauses with two noun phrases | 0.42 |  |  |
| Syntactic | Use of noun phrases with possessive pronouns and adjectives | 0.42 |  |  |
| Syntactic | Use of noun phrases with two noun phrases | 0.42 |  |  |
| Syntactic | Use of noun phrases with proper nouns | 0.42 |  |  |
| Acoustic | Mean of the 7th MFCC | -0.42 | 0.94 |  |
| Acoustic | Mean of the 5th MFCC | -0.42 | 0.96 |  |
| Acoustic | Mean of the 12th MFCC | -0.41 | 0.96 |  |
| Lexical | Vocabulary richness (TTR, ratio of unique words to total number of words, 30 word window) | -0.41 |  | 0.77 |
| Lexical | Vocabulary richness (TTR, ratio of unique words to total number of words, 20 word window) | -0.41 |  | 0.73 |
| Syntactic | Complexity of speech (undirected parallel edges in graph) | 0.40 |  |  |
| Acoustic | Mean of the 8th MFCC | -0.40 | 0.96 |  |
| Lexical | Vocabulary richness (TTR, 10 word window) | -0.40 |  |  |
| Syntactic | Use of noun phrases with determiners and nouns | 0.40 |  |  |
| Semantic | Complexity of speech (directed parallel edges in graph) | 0.39 |  |  |
| Acoustic | Skewness of the second derivative of the 3rd MFCC | 0.39 |  |  |
| Acoustic | Mean of the second derivative of the 1st MFCC | -0.39 | 0.97 |  |
| Lexical | Vocabulary richness (Brunet’s index) | 0.39 |  | -0.63 |
| Lexical | Use of subordinate words | 0.39 |  | -0.61 |
| Acoustic | Kurtosis of the second derivative of the 3rd MFCC | 0.38 |  |  |
| Acoustic | Mean of the first derivative of the 1st MFCC | -0.38 | 0.96 |  |
| Acoustic | Mean of the 1st MFCC | -0.38 | 0.97 |  |
| Acoustic | Variance of the second derivative of the 9th MFCC | 0.37 |  |  |
| Acoustic | Mean of the 9th MFCC | -0.37 | 0.98 |  |
| Acoustic | Mean of the first derivative of the 4th MFCC | -0.37 | 0.98 |  |
| Acoustic | Kurtosis of the second derivative of the 5th MFCC | 0.37 | -0.61 |  |
| Acoustic | Skewness of the second derivative of the 5th MFCC | -0.37 | 0.65 |  |
| Acoustic | Mean of the second derivative of the 0th MFCC | -0.37 | 0.95 |  |
| Acoustic | Mean of the first derivative of the 3rd MFCC | -0.37 | 0.99 |  |
| Acoustic | Mean of the first derivative of the 0th MFCC | -0.37 | 0.95 |  |
| Acoustic | Mean of the 0th MFCC | -0.37 | 0.95 |  |

*Columns F1 and F2 indicate which variables were assigned to each factor, and the factor loading scores. Variables with no values in the F1 and F2 columns correlated with errors in speech but were not included in a factor based on the exploratory factor analysis.*
